# Annual and Analytical Cyclopædia of Practical Medicine

**Published:** 1901-09

**Authors:** 


### Bibliography.

Annual and Analytical Cyclopædia of Practical Medicine. By Charles E. de M. Sajous, M.D., and One Hundred Associated Editors, assisted by Corresponding Editors, Collaborators, and Correspondents. Illustrated with Chromo-Lithographs, Engravings, and Maps. Vol. VI. F. A. Davis Company, Publishers, Philadelphia, New York, Chicago, 1901.

The editor says in his preface that "This volume is the last of the first series of the present work. . . . The work presents all the general diseases usually described in text-books, and besides what progressive features the past decade has furnished."

The very carefully prepared index gives a better idea of the magnitude of this entire work than even an examination of the six volumes could give, entering, as it does, into the minutiæ of the work, which the general reader will probably fail to do.

In the various reviews made of previous volumes of this series there could be but one favorable opinion expressed as each appeared. The present volume is large, one thousand and forty-three pages, including index, which covers one hundred and eight pages of double columns. In it are presented a series of valuable articles, equal, if not superior, to those that have preceded in previous volumes. Among these are "Rheumatism," by Dr. Levison, of Copenhagen; "Diseases of the Stomach," by Professor D. D. Stewart, of Philadelphia; "Surgery of the Stomach and Intestines," by Professor W. W. Keen and Dr. M. B. Tinker, of Philadelphia; "Surgery of the Spine," by Professor R. H. Sayre, of New York; "Syphilis," by Professor G. F. Lydston, of Chicago, etc.

There is nothing said in the prefatory notice in regard to a future edition except the expression "last of the first series," but it is presumed the subject matter will be added to from time to time and that which has become obsolete will be eliminated. The work as it stands now seems to the reviewer not only to cover the entire domain of medicine, but it does that so thoroughly that, if kept up to its present standard, it must be not only a reference work in the library, but an authority upon questions of pathology and therapeutics. The various writers have entered upon the discussion of diseases with a fulness not often met with in ordinary text-books, hence the special value of this cyclopædia. It has been an herculean task to prepare these volumes, and the scholarly editor and his assistants may be congratulated upon the successful completion of their work.

